# A criteria-based rehabilitation program for chronic mid-portion Achilles tendinopathy: study protocol for a randomised controlled trial

**DOI:** 10.1186/s12891-021-04553-6

**Published:** 2021-08-14

**Authors:** Colin Griffin, Katherine Daniels, Caroline Hill, Andrew Franklyn-Miller, Jean-Benoît Morin

**Affiliations:** 1 Université Côte d’Azur, LAMHESS, Nice, France; 2 Sports Surgery Clinic, Santry Demesne, Dublin 9, Ireland; 3 University of Bristol, Queen’s School of Engineering, University Walk, Bristol, BS81TR UK; 4 Department of Sport and Exercise Sciences, Manchester Metropolitan University, Manchester, UK; 5 Centre for Health, Exercise and Sports Medicine, University of Melbourne, Parkville, Victoria Australia; 6 Sports Performance Research Institute New Zealand (SPRINZ), Auckland University of Technology, Auckland, New Zealand; 7 Univ Lyon, UJM-Saint-Etienne, Inter-university Laboratory of Human Movement Biology, EA 7424, F-42023 Saint-Etienne, France

**Keywords:** Achilles tendinopathy, Plantar flexor strength, Stiffness, Hopping, Achilles tendon, Injury, Rehabilitation

## Abstract

**Background:**

Achilles tendinopathy (AT) is a common overuse injury in running-related sports where patients experience pain and impaired function which can persist. A graded rehabilitation program has been successful in reducing pain and improving function to enable a return to sport. The aim of this study is to compare the effectiveness of a criteria-based rehabilitation program including strength and reactive strength targets, with a previously successful rehabilitation program on changes in pain and function using the Victorian Institute of Sport Assessment-Achilles (VISA-A) questionnaire. Secondary aims will be to assess changes in calf strength, reactive strength, and lower limb running and forward hop biomechanics over the course of a 12-week rehabilitation program, and long-term follow-up investigations.

**Methods:**

Sixty eligible participants with chronic mid-portion AT who train in running-based sports will be included in this study. They will be randomly assigned to a group that will follow an evidence-based rehabilitation program of daily exercises with progression guided by symptoms or a group performing 3 high-intensity rehabilitation sessions per week with individualised load targets progressing to reactive strength exercises. Testing will take place at baseline, week 6 and 12. Plantar flexor peak torque will be measured using isokinetic dynamometry, reactive strength will be measured using a drop jump and lower limb biomechanical variables will be measured during a single leg forward hurdle hop test and treadmill running using 3D motion analysis. Follow-up interviews will take place at 6, 12 and 24 months after beginning the program which will assess patient participation in sport and possible re-injury.

**Discussion:**

This is the first study to propose an individualised criteria-based graded rehabilitation program in patients in with chronic mid-portion Achilles tendinopathy where progression is guided by strength and reactive strength outcome measures. This study will provide a comprehensive assessment of plantar flexor strength, reactive strength and lower limb biomechanical variables in running and forward hopping with the VISA-A questionnaire as the primary outcome measure and long term post-intervention follow-up assessments performed.

**Trial registration:**

ClinicalTrials.gov (ID: NCT04384874). Registered retrospectively on April 23rd 2020.

**Supplementary Information:**

The online version contains supplementary material available at 10.1186/s12891-021-04553-6.

## Background

The Achilles tendon is the largest and strongest tendon in the human body [1] and usually withstands very high tensile forces during exercise [2], but is also one of the most commonly injured tendons [3]. Achilles tendinopathy (AT) affects 2% of the general population [4], and has an incidence of 7–9% in running-based sports with a cumulative lifetime incidence of up to 52% among certain athletic populations [5].

Tendinopathy is described as pain and impaired function in the affected tendon [6–8]. Over time this may result in reduced physical activity, absenteeism from sport and impaired quality of life [8]. Histologically and biochemically pathological tendon has been shown to include increased hyper-cellularity, reduced collagen type I and increased type III content, increased proteoglycans and glycosaminoglycans, and blood vessel in-growth [9, 10]. Excessive loading of the tendon is believed to be the primary contributory factor to Achilles tendinopathy [11]. The fibroblastic cells known as tenocytes within the extracellular matrix are sensitive to mechanical loading and, when the tendon is overloaded, the cells alter the protein composition of the matrix resulting in pathology and reduced capacity for exercise [12].

Patients with AT usually present with pain, swelling and impaired performance of the tendon [13], as well as altered function of the plantar flexor muscles [14–16]. In a sporting population, training load perturbations such as a rapid increase in training volume, intensity or frequency are said to be common contributory factors [17]. Re-injury rates are high, most likely due to incomplete restoration of muscle-tendon unit function [18], and symptoms can persist for a number of years in some cases [19]. In an eight-year follow-up study, 20% of patients still experienced impaired physical activity [20]. A failed healing response and degenerative changes are associated with the development of chronic tendinopathy resulting in reduced load capacity and persistent pain symptoms [8]. This is described by Cook et al. [8] in their proposed continuum model involving three stages: reactive tendinopathy, tendon disrepair and degenerative tendinopathy.

Impairments in tendon mechanical properties such as stiffness and Young’s modulus have been highlighted in AT [21–23]. Wang et al. (2012) observed reduced stiffness and increased hysteresis of the Achilles tendon, along with reduced rate of force development of the plantar flexor muscles and reduced single leg horizontal hop performance in symptomatic limbs of patients with AT, compared to the non-symptomatic limb. While one study found reduced lower limb stiffness in the injured limb of runners with AT during hopping [24], biomechanical variables such as leg and joint stiffness in running and hopping tasks have not been extensively researched in patients with Achilles tendinopathy.

Many passive treatment therapies such as injections [25, 26], Non-Steroidal Anti-Inflammatory Drugs (NSAIDs) [10, 27], ultrasound [10, 27], shockwave [28, 29], laser [27], iontophoresis [25], acupuncture [30], orthotics [25, 29], wearing a night splint [31], vibration and cryotherapy [32], ﻿mucopolysaccharides [33], and a wait-and-see approach [28] have been used in the management of AT. However, there is strongest evidence for the use of exercise therapy as the primary treatment option [34–36]. Tendons adapt to exercise as the mechanical perturbation of the inter- and intra-fascicular cells triggers a molecular response signalling an expression of important proteins in the extra-cellular matrix which restore the mechanical properties of the tendon [37, 38]. Three main modes of exercise have been widely used, each associated with improvements in clinical symptoms to varying degrees, namely: Alfredson’s eccentric protocol [39], Silbernagel’s combined concentric-eccentric protocol [36] and the Heavy Slow Resistance protocol [40]. The Silbernagel protocol [see Table 1] comprises a combined concentric-eccentric exercise program performed daily, before progressing to plyometric exercises as symptoms permit, with no individualisation of exercise prescription and progression guided solely by pain symptoms on a numeric pain rating scale (NPRS).

**Table 1:** Silbernagel’s combined concentric-eccentric program

Phase 1: Weeks 1–2	
**Patient status:** Pain and difficulty with all activities, difficulty performing 10 single leg heel raises	
**Goal:** Start to exercise, gain understanding of their injury and of pain monitoring model	
**Treatment program:** Perform exercises every day:	
• Pain monitoring model information and advice on exercise activity	
• Circulation exercises (moving foot up and down)	
• Double leg heel raises standing on the floor (3 × 10–15 repetitions)	
• Single leg heel raises standing on the floor (3 × 10)	
• Sitting heel raises (3 × 10)	
• Eccentric heel raises standing on the floor (3 × 10)	
**Phase 2: Weeks 2–5**	
**Patient status:** Pain and difficulty with all activities, difficulty performing 10 single leg heel raises	
**Goal:** Start to exercise, gain understanding of their injury and of pain monitoring model	
**Treatment program:** Perform exercises every day:	
• Pain monitoring model information and advice on exercise activity	
• Circulation exercises (moving foot up and down)	
• Double leg heel raises standing on the floor (3 × 10–15 repetitions)	
• Single leg heel raises standing on the floor (3 × 10)	
• Sitting heel raises (3 × 10)	
• Eccentric heel raises standing on the floor (3 × 10)	
**Patient status:** Pain with exercise, morning stiffness, pain when performing heel raises	
**Goal:** Start strengthening	
**Treatment program:** Perform exercises every day:	
• Double leg heel raises standing on the edge of a step (3 × 15)	
• Single leg heel raises standing on the edge of a step (3 × 10)	
• Sitting heel raises (3 × 15)	
• Eccentric heel raises standing on the edge of a step (3 × 15)	
• Quick-rebounding heel raises (3 × 20)	
**Phase 3: Weeks 3–12 (longer if needed)**	
**Patient status:** Tolerates phase 2 exercise program well, no pain at distal portion of tendon, possibly increased or decreased morning stiffness	
**Goal:** Heavier strength training, increase or begin running and/or jumping	
**Treatment program:** Perform exercises every day with heavier load 2–3 times per week	
• Single leg heel raises standing on the edge of a step with added weight (3 × 10)	
• Sitting heel raises (3 × 15)	
• Eccentric heel raises standing on the edge of a step with added weight (3 × 15)	
• Quick-rebounding heel raises (3 × 20)	
• Plyometric training	
**Phase 4: Weeks 12–6 months (longer if needed)** **Patient status:** Minimal symptoms, morning stiffness but not every day, can participate in sports without difficulty	
**Goal:** Heavier strength training, increase or begin running and/or jumping	
**Treatment program:** Perform exercises every day with heavier load 2–3 times per week	
• Single leg heel raises standing on the edge of a step with added weight (3 × 10)	
• Eccentric heel raises standing on the edge of a step with added weight (3 × 15)	
• Quick-rebounding heel raises (3 × 20)	

Recent evidence suggests that magnitude of loading, irrespective of contraction mode, is the primary stimulus for tendon adaptation [41]. Isometric exercises using 5 × 45 s contractions at 70% maximal voluntary contraction (MVC) have been used for early management of tendinopathies with evidence suggesting an analgesic effect [42]. However, this has been since contested by the findings of O’Neill et al. [43]. Documented timeframes for rehabilitation interventions vary between 6 weeks to several months with no clear objective measures for return to sport. Patients with AT display impaired reactive strength qualities during hopping tasks and it is recommended to include plyometric training at an advanced stage of a rehabilitation program to prepare for the stretch-shortening cycle demands of running-based sports [19, 36]. A multi-stage rehabilitation program that includes the combination of strength development and plyometric training may thus be beneficial, but there is a lack of consensus on assessing these qualities to guide exercise prescription and progression through the rehabilitation pathway.

While numerous studies have shown positive clinical improvements and tendon adaptations to exercise [36, 39–41, 44], studies which investigate a periodised return to sport rehabilitation program with load targets and outcome measures for progression, are necessary due to the individualised nature of its initial presentation and diverse timeframes for recovery. The Sports Surgery Clinic (SSC) rehabilitation pathway [see Table 2] proposed in this study involves 6 stages of progressive rehabilitation (SSC6) from initial diagnosis and assessment, through developing strength, power and reactive strength, linear and multi-directional running, and return to performance. The existing literature has demonstrated positive clinical outcomes using Silbernagel’s rehabilitation program [18, 44] and we have selected this a suitable comparative control for this study which involves a graded progression pathway from combined concentric-eccentric exercises to plyometric training [36].

**Table 2:** SSC6 Rehabilitation program

Level 1 *Week 0–3* Entry criteria: Pain > 5/10 on SL calf raise	Kinetic chain (2 days per week) Box squat 3 × 8 reps @ 10RM Step-up 3 × 8 reps e/s @ 10RM	Calf Isometrics (daily) 5 × 45 s holds @ 60 s RM)		
Level 2 *Week 0–4* Entry criteria: Pain < 5/10 on SL calf raise	**Kinetic chain (2 days per week)** Front squat 4 × 8 reps @ 10RM Or Deadlift 4 × 8 reps @ 10RM Step-up 3 × 8 e/s @ 10RM Or Split squat 3 × 8 reps e/s	**Calf strength** (3 days per week) SL calf raises 4 × 8 reps @ 10RM *~ Begin with dumbbell and shoes off* *~ Aim to through 1st MTPJ and good rearfoot control* Seated SL calf raises 4 × 10 reps @ 12RM *~ Begin with kettlebell on knee and forefoot on a plate*		
Level 3 *Week 3–6* Entry criteria: Pain < 4/10 on SL calf raise	**Kinetic chain (2 days per week)** Front squat 4 × 6 reps @ 8RM Or Deadlift 4 × 6 reps @ 8RM Step-up 3 × 6 e/s @ 8RM Or Split squat 3 × 6 each side @ 8RM	**Calf strength** (3 times per week) SL calf raises 4 × 8 reps @ 10RM *~ Progress to a smith machine or barbell using rack for support* *~ Aim for > 70% BW* Seated SL calf raises 4 × 10 reps @ 12RM *~ Progress to smith machine or landmine press* *~ Aim for > 90% BW*	**Coordination/running technique** Ankling 3 × 10 m March 3 × 10 m A-skip 3 × 20 m	
Level 4 *Week 6–9* Entry criteria: Pain < 5/10 for 10 DL hops < 10% asymmetry in calf isokinetic tests	**Kinetic chain (2 days per week)** Front squat 4 × 6 reps @ 8RM Or Deadlift 4 × 6 reps @ 8RM Step-up 3 × 6 reps e/s @ 8RM Or Split squat 3 × 6 each side @ 8RM	**Calf strength** (3 times per week) SL calf raises 4 × 8 reps @ 10RM *~ Progress to a smith machine or barbell using rack for support* *~ Aim for > 80% BW* Seated SL calf raises 4 × 10 reps @ 12RM *~ Progress to smith machine or landmine press* *~ Aim for > 110% BW*	**DL Reactive strength** (2 times per week) DL Pogo hops in-place 4 × 10 (Day 1) *~ Keep knees straight and stiff* *~ Flat foot contacts* *~ Active dorsiflexion during flight phase* DL pogo hops forward 4 × 10 (Day 2) ~ *Keep knees straight and stiff* *~ Flat foot contacts* *~ Active dorsiflexion during flight phase*	
Level 5 *Week 9–12* Entry criteria: Pain < 4/10 for 10 SL hops Exit criteria: < 10% asymmetry in single leg vertical and horizontal RSI	**Kinetic chain (2 days per week)** Front squat 3 × 5 reps @ 7RM Or Deadlift 3 × 5 reps @ 7RM Step-up 3 × 5 reps e/s @ 7RM Or Split squat 3 × 5 each side @ 7RM	**Calf strength** (3 times per week) SL calf eccentric 4 × 8 reps @ 10RM *~ Use a smith machine/leg press/ barbell using rack for support* *~ Up on 2 legs, lower down slowly on 1 over 3 s* *~ Aim for > 100% BW or equivalent* Seated SL calf raises 4 × 10 reps @ 12RM *~ Progress to smith machine or landmine press* *~ Aim for > 110% BW*	**DL Reactive strength** (2 times per week) Drop jump 4 × 4 reps from 20 to 30 cm box ~ *Maximum jump height with minimal contact* *~ Minimal knee bend on ground contact* *~ Cue “imagine the floor is hot”*	**SL Reactive strength** (2 times per week) SL pogo hops in-place 4 × 10 e/s (Day 1) SL pogo hops forward 4 × 10 e/s (Day 2)
Level 6 *Week 12–26* Recommended maintenance program	**Kinetic chain (2 days per week)** Front squat 3 × 5 reps @ 7RM Or Deadlift 3 × 5 reps @ 7RM Step-up 3 × 5 reps e/s @ 7RM Or Split squat 3 × 5 each side @ 7RM	**Calf strength** (2 times per week) SL calf isometric 4 × 8 reps × 4 s holds *~ Use a smith machine/leg press/ barbell using rack for support* *~ Up on 2 legs, hold on 1* *~ Aim for > 140% BW or equivalent*	**DL Reactive strength** (2 times per week) Drop jump 4 × 5 reps from 20 to 30 cm box	**SL Reactive strength** (2 times per week) SL pogo hops in-place 4 × 12 e/s (Day 1) SL pogo hops forward 4 × 12 e/s (Day 2)

Abbreviations: DL - double leg, SL - single leg, reps - repetitions, e/s - each side, BW - bodyweight, RM - repetition maximum

Considering the multiple functional impairments experienced by athletes with AT, a battery of kinematic and kinetic tests to investigate plantar flexor strength, reactive strength and lower limb biomechanical variables in hopping and running, may provide guidance on exercise prescription, progression through a rehabilitation program and return to sport decision-making. To the best of our knowledge no study has assessed such breadth of athletic qualities affected by AT.

This study will aim to compare the outcome of SSC6, a multi-factorial, individualised criteria-based rehabilitation program with Silbernagel’s combined concentric-eccentric program, in physically active participants with chronic mid-portion AT. In addition to the commonly reported outcome measures of VISA-A, as secondary outcome measures we will assess plantar flexor strength, reactive strength and lower limb kinematic and kinetics during running and hopping at 6-week intervals during a 12-week rehabilitation program as these have not been reported previously. We also further aim to investigate the long term effects of rehabilitation programs and achieved outcome measures over a 6, 12 and 24-month follow-up period.

### Aims

Using the VISA-A questionnaire as the primary outcome measure, the aim of this study is to compare the effectiveness of Silbernagel’s daily exercise program with progression guided by pain symptoms, against SSC6’s exercise program carried out 3 times per week with specific load targets. We will assess plantar flexor strength using isokinetic dynamometry, reactive strength based on a drop jump, and lower limb biomechanics during a novel single leg horizontal rebound test and running, and investigate whether changes in these variables over the course of the 12-week rehabilitation program are associated with improved pain and function outcomes using the VISA-A questionnaire when comparing the two rehabilitation programs. We will assess participant satisfaction with their prescribed program, adherence and fidelity using a training diary and perform follow-up interviews at 6, 12 and 24 months to analyse participation in their sport and any potential re-injury rates.

## Methods

### Study design

This study will be a single-centre, parallel group randomized-control trial. The data collection will take place at the SSC Sports Medicine department at the Sports Surgery Clinic in Dublin. The study protocol has been reported using the Standard Protocol Items: Recommendations for Interventions (SPIRIT) statement guidelines [Available in supplementary files]. The study was approved by the Sports Surgery Clinic’s Research Ethics Committee, (Application number: SAREB13/05/19CG/MJ) and registered at ClinicalTrials.gov (ID: NCT04384874).

### Participants

Adult patients diagnosed with chronic mid-portion AT who participate in running-based sports will be invited to take part in this study. Patients who present to the Sports Surgery Clinic (SSC) with Achilles pain will be seen by a Sport and Exercise medicine physician, their history and clinical examination will be confirmed with magnetic resonance imaging (MRI). If the patient is diagnosed and meets the inclusion criteria, they will be invited to participate in the study and will be given an information sheet to read with a minimum of 24 h to consider before agreeing by signing a consent form [Additional file 2: Appendix 2]. Participants will also be recruited externally through adverts on social media channels, emails to coaching contacts and local sports clubs. Participants who feel that they are eligible and meet the inclusion criteria will be referred for examination by a sport and exercise medicine physician at the clinic to confirm diagnosis and eligibility for the study.

### Inclusion/exclusion criteria

#### Inclusion criteria

Participants will be eligible for this study if they are aged 18–45 years, perform running-based sports, are diagnosed with mid-portion AT, following a clinical examination by a sports medicine physician and confirmed with MRI, and have been experiencing symptoms for more than 3 months but less than 3 years.

#### Exclusion criteria

Patients will be ineligible for the study if they have a co-existing lower-limb injury, have had a running-related injury in the previous 12 months, or have had any peritendinous, or intra tendinous Achilles injection in the past 6 months, or previous Achilles surgery [15, 18, 44].

### Randomisation and blinding

Participants will be assessed at baseline before being randomly assigned to the intervention group or control group and will follow a prescribed rehabilitation program for 12 weeks. See Table 3 for a summary of the study design. The randomisation will be performed using the online tool www.sealedenvelope.com and the participant will be handed an envelope from an independent observer not involved with the study, containing their respective group allocation number. The principal investigator and training group investigators will be blinded to the group randomisation process. These procedures have been used in similar studies [45]. The participants will be prescribed with an exercise program with video demonstrations of each exercise under the supervision of the investigator in their respective group. The program will be carried out at home or in a local gym in addition to supervised rehabilitation sessions every 2–3 weeks at SSC. Testing will take place again at week 6 and 12. Follow-up interviews will take place at 6, 12 and 24 months after baseline testing. The investigator involved with the testing and follow-up interviews will also be blinded to the group allocation. The primary outcome measure will be changes to the VISA-A questionnaire. Secondary outcome measures will include plantar flexor strength, lower limb reactive strength, biomechanics and running gait.

**Table 3:** Overview of outcome measures over the course of the study

	Baseline	Week 6	Week 12	6 months	12 months	24 months
Body mass	X					
Body height	X					
Body mass index	X					
Sport/activity level	X			X	X	XX
VISA-A Questionnaire	X	X	X	X	X	X
Isokinetic plantar flexor peak torque (knee extension)	X	X	X			
Isokinetic plantar flexor peak torque (knee flexion)	X	X	X			
3D running gait analysis	X	X	X			
Double leg drop jump		X	X			
Single leg drop jump		X	X			
Single leg horizontal rebound		X	X			
Exercise compliance		X	X	X		

### Outcome measures and assessments

#### Investigations

At baseline, week 6 and 12, all participants will be required to complete a VISA-A questionnaire as well as perform isokinetic testing and 3D motion capture running gait assessment. In addition, hop testing will be performed at week 6 and 12 [see Table 3]. Hop testing is included in the testing battery from week 6 onwards as it is expected some participants with Achilles pain at baseline testing may be fearful of performing hopping tasks or risk of exacerbating their pain, and the data collected may not be an accurate reflection of their capabilities.

### Primary outcome measure

#### VISA-A questionnaire

The VISA-A questionnaire has been shown to be a valid, reliable and easy-to-use outcome measure tool for intervention studies on AT [46]. It consists of eight questions regarding pain and function during both daily living and sporting activities. The overall score is between 0 and 100 where higher scores represent reduced pain and improved function. An improvement of 21 points between 2 and 12 weeks of a rehabilitation program have been typically observed [44]. While the VISA-A score will not determine eligibility for inclusion into the study, it will be used to map progress over the course of the rehabilitation program and in the follow-up period. The difference in VISA-A score between both training protocols from baseline testing to the outcome testing at 6 weeks, 12 weeks, 3, 6 and 12 months; will formulate the primary outcome measure for this study.

### Secondary outcome measures

#### Isokinetic plantar flexor strength

Reduced plantar flexor strength is a common feature in patients with Achilles tendinopathy [14–16, 39]. One prospective study to date established that plantar flexor torque below 50 Nm was a risk factor for developing AT [47]. Isokinetic testing is commonly used to measure plantar flexor peak torque [15, 16, 39].

Two separate protocols will be used for this test. In the first protocol, the participant will lie prone with full knee extension. In the second protocol, the participant will lie supine with 80° knee flexion. When the knee is flexed to greater than 60°, the force contribution of the biarticular gastrocnemius muscles to plantarflexion is reduced, and is thus representative of the force produced predominantly by soleus muscle [48]. If similar plantar flexor peak torque deficits exist between the two protocols, the identified deficits may thus be attributed to the soleus muscle [15] which will influence exercise prescription.

The testing will be performed on a isokinetic dynamometer (Cybex Norm, Computer Sports Medicine Inc.). In both protocols, the participant will have their foot strapped to a pedal with the centre of axis of rotation aligned with the medial malleolus and a correction for gravity applied. Beginning with their uninjured limb, participants will be asked to perform a warm-up involving 5 sub maximal concentric plantarflexion and dorsiflexion contractions increasing progressively from 60 to 100% of their self-perceived MVC for familiarisation. The participants will then be required to produce a maximal plantarflexion force over 5 repetitions for 2 sets with a 1 min rest between sets. Verbal encouragement will be provided to produce maximal effort through full range of motion for each repetition. In the second test, the participants will lie in supine position with the knee flexed to 80° in order to specifically test the peak torque of the soleus. The same familiarisation protocol, sets and repetitions as the previous test will apply. Both tests will use an angular velocity of 60° per second and operate through an ankle range of between 30° plantarflexion and 20° dorsiflexion. Data will be sampled at 100 and peak torque expressed as percentage of body mass (Nm/kg %) will be reported on both limbs. Between-limb asymmetries in peak torque will also reported and analysed.

#### Three-dimensional running gait analysis

Altered running biomechanics and muscle recruitment strategies have been highlighted in runners with AT [49–52]. Using a proprietary three-dimensional optical motion analysis system (Run 3D, Oxford, United Kingdom) the following kinematic and spatiotemporal variables will be measured: contact time, aerial time, stride length, stride frequency and joint angular displacements from initial contact to mid stance phase. Lower limb stiffness will be calculated using a validated equation based on the spring-mass model with running speed, contact time, body mass and leg length as inputs [53]. The participants will warm-up by running for between 2 and 5 min on the treadmill at a self-selected speed. Once they report that they are adequately warmed up they will be instructed to run at a speed that they feel they would be comfortable running at a steady pace for 30 min. Data will be captured for 30 s at a random interval over a 2 min period and the participants will not be informed about when the data capture begins. For the subsequent tests at week 6 and 12, the participants will be required to repeat the same speed for re-analysis.

#### Hop tests

Achilles tendon material properties contribute to stretch shortening cycle performance during hopping and jumping exercises [54, 55]. Reduced tendon mechanical properties, plantar flexor muscle rate of force development and deficits on a single forward hop test have previously been observed in patients with AT [23].

The hop tests will take place on two force platforms (AMTI, USA) to measure ground reaction force (GRF) data sampled at 1000 Hz. Ten infrared cameras (200 Hz; Bonita B10/Vero v2.2, Vicon, UK) will be used for three-dimensional motion capture. Reflective markers (14 mm diameter) placed on all relevant anatomical landmarks including the thorax, will be used in accordance with a modified Plug-in-gait model (Vicon, UK) [56], with centre of mass (COM) calculated from all segments. Motion and force data will be filtered using a fourth order zero-lag low pass Butterworth filter with a cut-off frequency of 15 Hz. The data will be exported to MATLAB 2015a (Mathworks, USA) for processing. Participants will perform 3 trials on each test, unshod and with hands placed on iliac crests.

##### Drop jump

Participants will perform both a double leg drop jump (DLDJ) and single leg drop jumps (SLDJ). The participants will complete a standardised warm-up which consists of 10 bodyweight squats, followed by 10 pogo hops in place and 3 familiarisation trials for both. A 30 cm box will be used for the DLDJ and a 20 cm box for the SLDJ. The participant who will be unshod with hands placed on iliac crests, will be required to drop off the box and rebound off the force plate as quickly as possible aiming for maximum jump height. They will be instructed to maintain knee and hip extension during flight phase and where there is visible evidence of knee flexion or a ‘tuck jump’, the trial will be deemed invalid and they will be asked to repeat until a competent trial is achieved. The ground contact phase will be defined by a GRF greater than 20 N and jump height will be calculated from centre of mass displacement using kinematic data. Reactive strength index (RSI), which is a measure of jump height divided by ground contact time, will be calculated for both the double and single leg drop jump.

##### Single leg hurdle hop

After completing the drop jump tests, participants will be asked to perform a single leg forward hurdle hop test (SLHH). The test requires that participants to perform a single leg forward hop over two 15 cm hurdles rebounding off the force platform in between, completing 3 trials on each leg. The participants will be instructed to rebound ‘as fast as possible’ and ‘as far as possible’, and to attempt to be fully stable on 1 leg upon landing. After each trial the participants will walk back slowly to begin the next trial taking approximately 10 s recovery time. Hop distance, rebound distance and contact time, as well as key biomechanical variables such as vertical, horizontal and leg ground reaction force, vertical, leg and joint stiffness, joint powers and moments, and joint angular displacements will be calculated using a custom MATLAB script (Mathworks, USA). Hop distance will be calculated as the distance from the initiation of the hop to the initial contact as the participants lands at the end of the hop and rebound distance from the force plate to the landing. Vertical stiffness (K_vert_) will be calculated at the point of maximum displacement of COM, as the ratio of change in vertical ground reaction force (GRF) to COM displacement:

$$ {\mathrm{K}}_{\mathrm{vert}}=\Delta \mathrm{Force}/\Delta \mathrm{CoM} $$

Leg stiffness will be calculated in the sagittal plane as the ratio of change in leg ground reaction force (F_leg_) to the change in leg length at the shortest leg length during stance phase as previously proposed [57]. Leg length is measured as the distance from the hip joint centre to the centre of pressure in the sagittal plane, while F_leg_ is calculated from the resultant GRF magnitude scaled to the leg angle using the trigonometry sine rule.

$$ {\mathrm{K}}_{\mathrm{leg}}=\Delta {\mathrm{F}}_{\mathrm{leg}}/\Delta \mathrm{Leg} $$

Joint stiffness (K_ankle_, K_knee_ and K_hip)_ at the ankle and knee, will be calculated in the sagittal plane as the ratio of change in joint moment to change in joint displacement:

$$ {\mathrm{K}}_{\mathrm{joint}}=\kern0.5em \Delta \kern0.5em \mathrm{moment}/\Delta \kern0.5em \mathrm{angle} $$

A pilot study has previously been carried out on 10 healthy participants prior to the commencement of the Achilles RCT study [58]. Good-to-excellent reliability (ICC > 0.75) was found for hop and rebound distance, contact time, knee and ankle joint stiffness, vertical and leg GRF, with moderate reliability (ICC 0.50–0.75) for reactive strength index, vertical and leg stiffness, ankle joint peak power, ankle and knee joint peak moments, and horizontal GRF. In a separate study using the same protocol, 3 trials were sufficient to obtain a stable measure of performance across key variables [59].

#### Training diary

In order to determine adherence and fidelity with the rehabilitation program and pain response to exercise, each patient will be required to complete a training diary logging their completed running and rehabilitation sessions as well as reporting any pain symptoms using a numerical pain rating scale (NPRS), that will be reviewed at week 6 and returned at week 12. Adherence is defined as the proportion of prescribed exercises completed while fidelity refers to whether the participant completed the prescribed exercises, sets, repetitions and target loads. Participants will be advised to take an extra recovery day between exercise sessions if pain was above 5/10 on the day after a session and to adjust their loads for the subsequent session.

#### Follow-up interviews

**Fig. 1:**
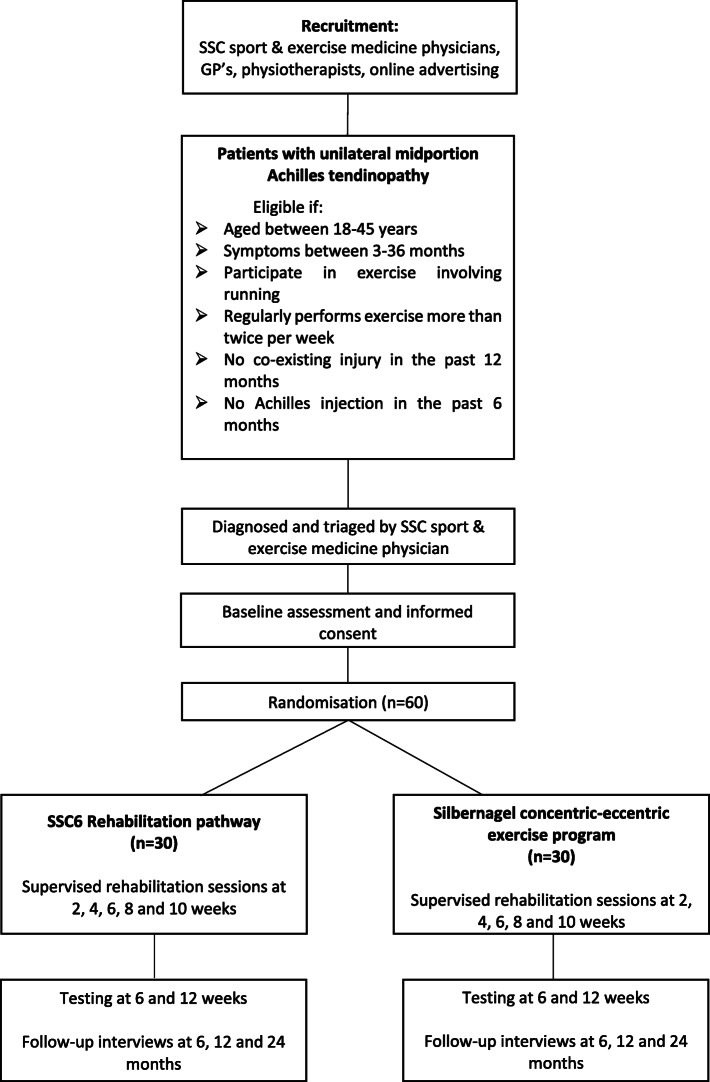
Study design flow chart

At 6, 12 and 24 months from baseline testing, patients will be required to complete a questionnaire [see Additional file 1: Appendix 1] to analyse their participation in their respective sport, document any re-injuries and to obtain patient satisfaction feedback on their respective rehabilitation program. These outcomes will be reported and compared between groups to determine if the rehabilitation program had any significant effect (Fig. 1).

### Interventions

Each participant will be prescribed a graded rehabilitation exercise training program which they will perform at home or in a local gym. However, they will present themselves for one supervised session every 2–3 weeks by their respective group investigators to ensure compliance and appropriate progression. The patients following Silbernagel’s training program [see Table 1] will perform solely calf strength exercises with self-prescribed additional resistance and will progress their exercises based on a NPRS, where there is no greater than a 4/10 pain response during and in the 24 h following a training session. They will then progress to plyometric exercises as tolerated. The SSC6 group [see Table 2] will follow a multifactorial exercise program comprising of bilateral and unilateral kinetic chain strength, calf strength and plyometric training as well as running drills as early as they can tolerate them. The participants will enter at the highest level where they meet the minimum criteria. For the calf exercises, they will be encouraged to lift a certain percentage of bodyweight in additional resistance and increase weekly. A certain level of pain within tolerable limits will be accepted and participants will be encouraged to increase their resistance loading weekly so long as that pain doesn’t increase. Progression to Level 4 of the program will be based on achieving their prescribed exercise load targets and achieving a deficit of less than 10% between injured and uninjured limbs on the isokinetic strength tests. The reactive strength exercises will be performed at near maximal intensity for a set number of repetitions with good competency and within tolerable pain limits. The participants will progress to Level 5 when they can perform 10 single leg hops with a score of < 4/10 on the NPRS and progress from Level 5 when single leg RSI deficits are < 10%. Outcome measures will be monitored at the various timepoints and will be tracked according to reported NPRS ratings. In both groups, participants will be permitted to begin running in phase 2 when pain during daily activity is < 2/10 but will be advised on periodising their running and rehabilitation exercises throughout the week. Each participant will be provided with a training log in order to monitor training loads. Should an adverse event occur which results in re-injury or a new injury, the participant will be instructed to contact their respective investigator immediately so that they can be examined and their treatment will be adjusted, postponed or discontinued where appropriate. Upon completion of the training intervention, participants in both groups will be given a maintenance training program for 6 months. The design, prescription and reporting of the training intervention meets all of the 16-item checklist requirements in the Consensus on Exercise Reporting Template (CERT) [60] [Available in supplementary files].

See Tables 1 and 2 for an example of the exercise programs, and Table 4 for the points of difference between to two training interventions.

**Table 4:**
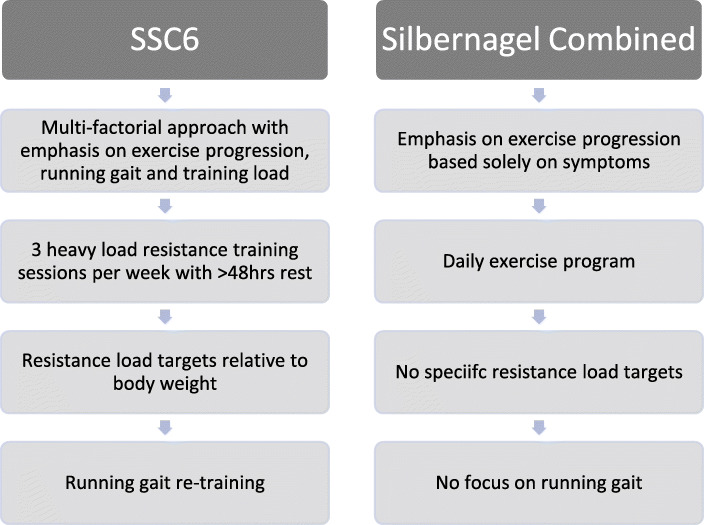
SSC6 Versus Silbernagel’s rehabilitation program.

### Statistical analysis and power calculation

This study is powered to detect a change of 15 points on the VISA-A questionnaire, similar to previous studies investigating clinical changes after a rehabilitation program [61, 62]. The average reported change in a VISA-A questionnaire after a 12-week intervention is 21 points with a standard deviation 6.6 points [44]. Assuming a power of 80% and a two-sided 5% significance level, a total of 25 participants in each group would be required. Allowing for a conservative drop-out rate of 15%, the proposed total sample size is 60, with 30 in each group. This number is similar to those used in other high quality injury rehabilitation RCT studies [40, 45].

Statistical analysis will be performed using R (R Studio version 1.2.5). Descriptive statistics will be used for all continuous variables, and means and standard deviations will be reported. Comparisons between both groups at different timepoints will be assessed using Student’s independent samples two-tailed t-tests. An intention to treat analysis will be used to test a within-group and between-group change in VISA-A questionnaire score at testing and follow-up timepoints, using a repeated measures Analysis of Covariance (ANCOVA). The primary outcome measure - changes to the VISA-A questionnaire, will the dependent variable, group will the between participants factor and time will be the covariate. Non-parametric equivalents (Matt-Whitney U-Test and Friedman Test respectively) will be used if a Shapiro-Wilk test indicates that the assumption of normality has not been met. A multiple regression analysis will be used to investigate the total variance and the relative weight of each independent variable with changes in VISA-A score as the dependent variable. The independent variables will be changes in plantar flexor strength, reactive strength and lower limb biomechanical variables, as well as exercise adherence and fidelity. Effect sizes will be reported using partial eta squared threshold values of > 0.2 (small), > 0.5 (moderate), and > 0.8 (large). Statistical significance will be accepted at α = 0.05.

## Discussion

Exercise therapy is widely accepted as the primary treatment option for runners with AT [35, 36, 63]. Heavy resistance strength exercises targeting the muscle-tendon unit have been shown to increase physiological cross-sectional area and pennation angle in the muscle [64] and tendon mechanical and material properties [41, 65]. This type of loading has resulted in improved clinical outcomes in AT patients [40, 44]. Plyometric training prepares the muscle tendon unit for high tensile forces and loading rates associated with running based sports [2, 36, 66]. However, there is no clear guidance on how to prescribe and progress the loading for calf strength exercises apart from using pain response to exercise. Only a few studies have investigated running biomechanical features associated with AT with limited evidence for poor control of rearfoot eversion [50, 67] and reduced leg stiffness on the injured limb [24].

An acceptable level of pain symptoms are permitted during AT rehabilitation [18, 34] and it remains to be explored if a primary focus on achieving strength, reactive strength and biomechanical targets can lead to similar outcomes in reduced timeframes and with lower re-injury rates. This is the first study to propose an individualised, criteria-based graded rehabilitation program in patients in with chronic mid-portion Achilles tendinopathy where progression is guided by strength and reactive strength outcome measures within tolerable pain limits. The participants in our study will undertake a comprehensive assessment of kinematic and kinetic tests to investigate plantar flexor strength, reactive strength and lower limb biomechanical variables in hopping and running. We will perform long term evaluations at evaluations at 6, 12 and 24 months to monitor progress, re-injury incidences and sustainability of return to sport and investigate patient satisfaction with their respective rehabilitation exercise programs.

Our study will include a sample of participants who practise running-based sports and are of a particular age profile (age 18–45), have had no injection therapies in the previous 6 months and no co-existing lower limb injuries. Like most studies of similar design, there is a high risk of drop-outs, poor compliance with the respective programs and failure to respond to the follow-up questionnaires. This will be managed by aiming for a higher number of participants than the study is powered for and maintaining regular communication with the participants.

In summary, this two-arm RCT will compare the effectiveness of a criteria-based rehabilitation program with progression guided by achieving functional outcome measures with an evidenced-based program where progression is guided solely by pain symptoms. The results of this study will provide insights as to whether improved strength, reactive strength and lower limb biomechanics are associated with reduced pain in patients with chronic mid-portion AT and assist clinicians treating this injury to set objective criteria to progress rehabilitation and return to sport.

### Trial status

Recruitment for the trial started in January 2020 and it is anticipated that data collection will be completed in April 2023. As of November 9th 2020, 18 participants have been included.

## Supplementary Information

**Additional file 1.** Appendix 1: Follow-up questionnaire at 6 months.

**Additional file 2.** Appendix 2: Consent form.

## Data Availability

Data sharing is not applicable to this article as no datasets were generated or analysed during the current study.

## Abbreviations

ANCOVA: Analysis of covariance
AT: Achilles tendinopathy
CERT: Consensus on Exercise Reporting Template
COM: centre of mass
DLDJ: double leg drop jump
GRF: ground reaction force
ICC: intraclass correlation
K: stiffness
MRI: magnetic resonance image
MVC: maximal voluntary contraction
NPRS: numerical pain rating scale
NSAID: non-steroidal anti-inflammatory drug
RCT: randomised controlled trial
RSI: reactive strength index
SLDJ: single leg drop jump
SLHH: single leg hurdle hop
SPIRIT: Standard Protocol Items: Recommendations for Interventional Trials
SSC: Sports Surgery Clinic
VISA-A: Victorian Institute of Sports Assessment - Achilles
