# Supplementary material for: A criteria-based rehabilitation program for chronic mid-portion Achilles tendinopathy: study protocol for a randomised controlled trial
Source: BMC Musculoskelet Disord. 2021 Aug 14;22:695. doi: 10.1186/s12891-021-04553-6 (PMC8364697; doi:10.1186/s12891-021-04553-6)
Supplement: Supplementary file 1 — Additional file 1. Appendix 1: Follow-up questionnaire at 6 months. [file 12891_2021_4553_MOESM1_ESM.docx]

**Appendix 1: Follow-up questionnaire at 6 months**

1. How would you rate your satisfaction with the rehabilitation program?

Poor Moderate Good Excellent

v
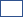


v
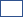


v
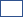


1. Are you likely to continue with the exercise program for the foreseeable future?

Very unlikely Unlikely Possibly Very likely

1. What was your average weekly training hours over the following timepoints:

Immediately prior to commencing the study?

Over the 12 weeks of the rehabilitation program?

From week 12-26?

1. Since beginning your rehabilitation program, how would you describe the change (if any) in Activity Limitations, Symptoms, Emotions, and Overall Quality Of Life related to your Achilles tendon injury?

No change (or condition has got worse) 1

Almost the same, hardly any change at all 2

A little better, but no noticeable change 3

Somewhat better, but the change has not many any real difference 4

Moderately better, and a slight but noticeable change 5

Better, and a definite improvement that has made a real and worthwhile difference 6

A great deal better, and a considerable improvement that has made all the difference 7

1. Can you describe the positive aspects (if any) about your rehabilitation program (open question)?
2. Can you describe any negative aspects about your rehabilitation program (open question)?
