# Supplementary material for: A criteria-based rehabilitation program for chronic mid-portion Achilles tendinopathy: study protocol for a randomised controlled trial
Source: BMC Musculoskelet Disord. 2021 Aug 14;22:695. doi: 10.1186/s12891-021-04553-6 (PMC8364697; doi:10.1186/s12891-021-04553-6)
Supplement: Supplementary file 2 — Additional file 2. Appendix 2: Consent form. [file 12891_2021_4553_MOESM2_ESM.docx]

**Appendix 2: Consent form**

Unit C10, Gulliver’s Retail Park,

Northwood,

Santry,

Dublin 9

www.sportssurgeryclinic.com


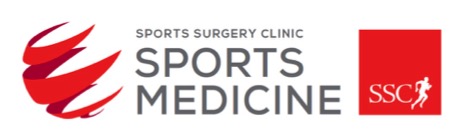


**A new multi-factorial, individualised rehabilitation program for chronic mid-portion Achilles tendinopathy**

**Consent to take part in this research study**

- I [print name] ………………………………………………… voluntarily agree to participate in this research study.
- I understand that even if I agree to participate now, I can withdraw at any time up to two weeks after my last testing session. If I withdraw from the study but still wish to continue my treatment, I will be charged for my physiotherapy sessions at our regular rate thereafter.
- I have had the purpose and nature of the study explained to me in writing and I have had the opportunity to ask questions about the study and given a minimum of 24 hours to consider taking part.
- I understand that participation involves partaking in a guided exercise program and making myself available for 3 testing sessions that may each last up to 2 hours over a 12-week period, and to be available for a brief interview at 6, 12 and 24 months upon completion of the study.
- I understand that if I were to miss any of the three scheduled testing sessions, I would no longer be eligible for the study.
- I understand that if I inform the researcher that I am at risk of injury or harm, they may have to refer me to a sport and exercise medicine physician or physiotherapist at the Sports Surgery Clinic who may decide that I am no longer suitable to continue with this study.
- I understand that signed consent forms, original video recordings and all data collected will be retained in the Sports Surgery Clinic under password protection for up to 3 years and only accessible to the principal investigators and research staff. After this period the data will be destroyed by the principal investigator.
- I understand that under the freedom of information legislation I am entitled to access the information I have provided at any time while it is in storage as specified above.
- I understand that I am free to contact any of the people involved in the research to seek further clarification and information.

*Signature of research participant*

----------------------------------------- ----------------

Signature of participant Date

*Signature of researcher*

------------------------------------------ ------------------

Signature of researcher Date
